# The effect of an intervention for organizing primary mental health care in Brazil: a study based on routine process indicators

**DOI:** 10.1186/s12875-025-02735-y

**Published:** 2025-02-26

**Authors:** Ilana Eshriqui, Letícia Yamawaka de Almeida, Joana Moscoso Teixeira de Mendonça, Leandro Anazawa, Lorrayne Belotti, Sandra Fortes, Ana Alice Freire Sousa, Evelyn Lima de Souza, Claudielle de Santana Teodoro, Daiana Bonfim, Joel de Almeida Siqueira Junior, Antonio Cleilson Nobre Bandeira, Lucas Reis Correia

**Affiliations:** 1 Hospital Israelita Albert Einstein, São Paulo, SP Brazil; 2 Faculty of Medical Sciences, University of the State of Rio de Janeiro, Rio de Janeiro, RJ Brazil; 3 Federal University of Santa Catarina, Florianópolis, SC Brazil

**Keywords:** Mental health, Primary care, Health care planning, Health care evaluation

## Abstract

**Background:**

The “Mental Health Care in Primary Health Care (PHC)” project (SMAPS, from Portuguese) was developed in six health regions from three Brazilian states. Considering the gap and relevance of monitoring and assessing mental health (MH) care in real-world settings using data from service records, this study aimed to evaluate the effect of SMAPS on indicators of MH care process in PHC, calculated from official records available in health services.

**Methods:**

This is a pilot study conducted between January 2022 and September 2023. The sample comprised 18 PHC units in 14 municipalities, which were in one of the three Brazilian states that adhered to SMAPS and assigned to one of two groups: control and intervention (2:1). Secondary data were collected at two times using the same instrument to systematize the retrieval of aggregated data, which were extracted from management reports from PHC units or municipalities departments. Data analysis was performed using descriptive statistics at the PHC units or municipality level.

**Results:**

The rate of referrals to MH specialties per consultation with a MH classifications record seemed to decline in intervention and control group. The percentage of benzodiazepine medications delivered for PHC decreased from 16.1 to 11.3% in the intervention group municipalities but increased from 20.3 to 24.1% in the control group. An inverse pattern was observed for antipsychotic, anticonvulsant, and antidepressant medications (increased from 82.2 to 86.2% in the intervention group municipalities and declined from 77.6 to 74.4% in the control group). Despite this, analyzing the mean variation in MH care indicators, statistically significant differences between groups in distribution at the 5% level were not identified.

**Conclusion:**

The present pilot study points to possible effects of SMAPS for organizing MH care in PHC, especially regarding the referrals to specialty mental health care and the delivery of psychotropic medications. It contributes to the formulation of proposals for evaluating MH care based on data already available from records and reports from health services and emphasizes the need to improve the quality of MH care recording in PHC work routines.

## Introduction

The epidemiological estimates of mental and substance abuse disorders globally underscore the great challenge confronting health systems in their quest for improved outcomes. This challenge stems from the necessity to allocate finite resources and to devise innovative measures in the face of the burden of diseases, their consequences, and the substantial gaps in treatment [1, 2].

The current recommendations of the World Health Organization (WHO) indicate [3], among other aspects, that primary health care (PHC) is a strategic component of care in terms of access, identification, and treatment of people in mental distress. Thus, growing efforts have been reported in the literature regarding the integration of mental health (MH) care in PHC [4, 5]. In contrast, some obstacles related to stigma [6], the need for professional training and qualification [7], and organization of work flows and processes [7, 8] have been observed.

For the initiatives related to low- and middle-income countries, those guided by the Mental Health Gap Action Programme (mhGAP) proposed by the WHO [9] deserve special recognition. The evidence gathered in a recent systematic review [3] describes promising results from programs focusing mainly on countries in Africa and Southeast Asia, signaling a gap in studies that evaluate and investigate proposals in other regions.

In Brazil, recent efforts such as the project “Implementation of Mental Health Care in PHC for the organization of the Network” (referred to as Mental Health in Primary Health Care- SMAPS, from Portuguese “*Saúde Mental na APS*”) have stood out as an initiative supported by the Brazilian Ministry of Health and the National Council of Health Secretaries (CONASS), implemented in real PHC settings in the Brazilian Unified Health System (SUS: Sistema Único de Saúde). The project SMAPS trains non-specialized professionals from the *SUS* for the identification and management of priority MH conditions in addition to strengthening work processes in this care setting.

Brazil’s Unified Health System (SUS) operates through an interfederative arrangement involving the Ministry of Health, state governments, health regions, and municipalities. The Primary Health Care (PHC) model of SUS is the Family Health Strategy, where services are delivered by municipalities, which correspond to counties in other contexts. These municipalities are grouped into health regions at the state level, allowing for more effective planning and implementation of stepped care. The PHC units are organized based on the population they serve within their respective municipalities, which are then integrated into the broader framework of health regions to promote comprehensive, equitable, and well-coordinated care.

The proposal for SMAPS, which has been implemented in six health regions within three Brazilian states, seeks to support municipal and state governments in organizing MH care delivery in PHC, based on the combination of two multifaceted implementation strategies — the Health Care Planning (HCP) methodology and training for the use of the mhGAP intervention guide (mhGAP-IG) [10, 11].

The HCP is a methodology designed to enhance the competence of healthcare teams in planning, organizing, and monitoring work processes, with a focus on patient’s needs. It incorporates a robust pedagogical component to develop the knowledge, skills, and attitudes necessary for delivering good quality health care. Its main goal is to prepare teams for the effective operationalization of health care networks, emphasizing several priority lines of care. In SMAPS, the combination of HCP and mhGAP-IG training strategies aims to strengthen PHC competences to effectively organize MH care [10].

Operationalized in four stages, the SMAPS intervention was provided to all PHC professionals in each participating municipality, ensuring full inclusion of all health care workers. It comprised a workshop for theoretical alignment and tutorial sessions that combined discussions and practical tools to transform team and service operations. Key themes addressed were the role of PHC within the Psychosocial Care Network, territory and population-based management, access, and care management.

The SMAPS project was designed to address mental health equitably within communities through territorialization and risk stratification, enabling early detection and targeted prevention by mapping family risk and identifying warning signs during community health workers (ACS: Agente Comunitário de Saúde) from home visits. Efforts to ensure equitable access involved analyzing the demand and supply of mental health care in health units and organizing service schedules based on the demographic and health conditions of each territory. The approach emphasized population-based management, training PHC professionals in stepped care, enhancing the “Matrix support” model, and integrating psychosocial interventions in partnership with other sectors, such as culture, leisure, and education, extending mental health promotion into broader community contexts. To further clarify the components of the intervention, we build on the methodological approach outlined by Eshriqui et al. (2023) in their article “Using implementation science to evaluate mental health intervention: methodological proposal” [11]. 

It is worth noting that, from a managerial perspective, SMAPS emphasizes the importance of monitoring indicators given the limited availability of epidemiological data regarding the population with mental health conditions at the state, municipal, and service levels within the respective regions. In this scenario, considering that systems of official records have fields to fill in data that allow for the calculation of mental health care indicators, SMAPS provides for technical and operational materials to support the incorporation of recording and monitoring of mental health care indicators, such as suicide rates, rate of referrals to specialty mental health care, and psychiatric admissions, into the routine of health services. Also, considering that in Brazil there is a concern regarding the misuse of benzodiazepines, especially among those diagnosed with major depression and generalized anxiety disorder [12–14], which could be benefit from use of other classes of psychotropics, such as antidepressants, it is important to establish indicators viable to be used into service and management routines to monitor the pattern (not only the amount) of psychotropics medications prescriptions.

Thus, considering the significance of monitoring and assessing program outcomes in real-world settings using data from service records [15–17] to inform decision-making, and to improve the quality of mental health care, and contribute to the ongoing discourse in the literature, this study aimed to evaluate the effect of SMAPS on indicators of mental health care process in PHC, calculated from official records available in health services.

## Methods

### Type of study

This quasi-experimental study evaluated the effect of the SMAPS project by observing its implementation in selected municipalities and comparing outcomes with control municipalities that shared similar characteristics. The study was conducted between January 2022 and September 2023 and received ethical approval from the Albert Einstein Hospital Ethics Committee (CAAE: 12395919000000071).

The SMAPS intervention, described in detail in previous studies [10, 11], establishes among its implementation strategies, the HCP methodology, through four operational stages, in addition to stages for alignment and planning with stakeholders; and training of multipliers and all PHC professionals (Physician, nurse, nursing assistant, community health workers, and multiprofessional team, such as psychologist, psychiatric, nutritionist, dentistry, physiotherapy, social assistant) for the use of mhGAP-IG. During SMAPS, 1,091 PHC professionals from the six participating health regions distributed across three states from the Northeast, North, and Central-West regions of Brazil, received training, as follows: 200 in Health Region 1 (Northeast region), 101 in Health Region 2 (North), 37 in Health Region 3 (North), 350 in Health Region 4 (Central-West), 312 in Health Region 5 (Central-West), and 91 in Health Region 6 (Central-West).

HCP operationalization throughout the SMAPS stages comprised a series of activities, including tutoring activities with all PHC professionals, managerial activities with stakeholders, as well as multidisciplinary discussions on the implementation of patient safety centers. In order to systematize and support these activities, SMAPS provides technical materials, offers distance learning courses, and the institutional support, providing ongoing consulting from external professionals.

### Selection procedure

The study employed a convenience sample selected based on specific criteria. The sample comprised 18 PHC units located in 14 municipalities, which were assigned to one of two groups: intervention (SMAPS project) and control.

To select the intervention (SMAPS project), the State Health Departments of the Brazilian states where SMAPS has been implemented were contacted to assist with sample selection. One PHC unit from each of the six health regions that adhered to SMAPS was invited to join the intervention group (SMAPS project) (*n* = 6 PHC units). The decision for these six health regions to adhere to SMAPS project (including 62 municipalities and 340 PHC units) was made through political agreements between the Ministry of Health and the respective state governments, rather than being influenced or determined by the researchers. The selection of specific PHC units for the intervention group followed the criteria established by the researchers: (i) the unit was a laboratory unit of SMAPS, serving as a showcase for other PHC units in the Health Region; (ii) it was located in an urban area with easy access; (iii) municipal PHC coverage was close to or greater than 80%; and (iv) it demonstrated greater progress in HCP implementation status according to a diagnostic assessment conducted between May and July 2022.

To characterize the participating health regions of SMAPS based on their adherence to health services and the implementation of HCP interventions, the “SMAPS HCP–Performance Index” (iHCP–Performance) [18] was developed by the SMAPS team as an operational index for the project. Table 1 describes the profile of the health regions using the iHCP-Performance in 2023, providing context for the study scenarios in which indicators related to MH care process were collected and measured. An index score of 7.0 or higher is considered satisfactory.

**Table 1:** Characteristics of the Health Care Planning (HCP) Index. SMAPS iHCP–Performance, Brazil, 2023

Health Regions	iStructure	iManagement	iTutoring	iQuality	iEducational	iHCP Performance
Health Region 1 (Northeast)	8.5	8.8	9.3	9.2	8.9	**9.0**
Health Region 2 (North)	9.5	8.3	7.5	6.7	5.7	7.8
Health Region 3 (North)	10.0	8.3	7.7	2.2	3.9	7.4
Health Region 4 (Central-West)	9.6	9.2	8.9	9.3	6.6	8.9
Health Region 5 (Central-West)	10.0	8.8	9.4	8.8	6.5	**9.0**
Health Region (Central-West)	9.4	8.3	9.1	6.2	6.6	8.4
SMAPS	9.6	8.6	8.8	8.5	7.2	8.7

Note: iStructure: registration of laboratory and expansion units registered in e-Planifica (weight 2). iManagement: Planning and Monitoring Workshops held at state and regional levels in the planned stages (weight 3). iTutoring: implementation of activities involving the tutoring process: Pre- and Post-Tutoring Alignments, Workshops, and participation by health units in Workshops and Tutoring Workshops in the planned stages (weight 4). iEducational: registration and entry of students and their approvals in management and tutoring refresher courses by Technical References and Tutors, respectively, as well as training of professionals in mhGAP (weight 1). iQuality: implementation of the State Working Group and meetings of the Patient Safety Center (PSC) at the state level; and implementation of the municipal PSC and Safety Team (weight 1). iHCP Performance = (iStructure × 2) + (iManagement × 3) + (iTutoring × 4) + (iQuality × 1) (iEducational × 1) / 11

For each of the six intervention PHC units, two PHC units (*N* = 12) were selected as controls, according to the following similarity criteria with the respective intervention PHC unit: (I) located in the same state, but in a different health region not participating in SMAPS; (II) located in an urban area with ease access; (III) municipal PHC coverage; (IV) number of family health teams; (V) population registration coverage of the territory covered by the unit; (VI) status of HCP implementation at baseline according to previously held workshops and thematic tutorial workshops. Additionally, participation of the health region and respective PHC unit in the control group was conditional on no mental health interventions being underway in the health region.

### Data collection

The study exclusively utilized secondary data retrieved from the SUS (Brazil’s Unified Health System) database, specifically the Electronic Citizen Record (ECR) system provided by the e-SUS platform and pharmacy report extracted from local information system. These databases are routinely updated by PHC professionals and managers to support health monitoring at both local and national levels. By leveraging this existing infrastructure, the study aimed to evaluate the SMAPS project’s effect using standardized data that reflect routine health care delivery. This approach also demonstrates the potential for utilizing nationally viable indicators to monitor mental health care in PHC settings, contributing to the scalability and sustainability of such evaluations across Brazil.

Secondary data were collected at two times. Baseline was established using data collected between August and September 2022, in the initial stage of the SMAPS Project. The theoretical discussion on the topic of mental health in PHC and the diagnosis of HCP and the Psychosocial Care Network (PSCN) in the participating health regions was then initiated. The second data collection point was between August and September 2023, during the beginning of the last operational SMAPS stage. Thus, for reasons of research feasibility, data collection was performed before the end of the intervention, scheduled for December 2023.

**Fig. 1:**
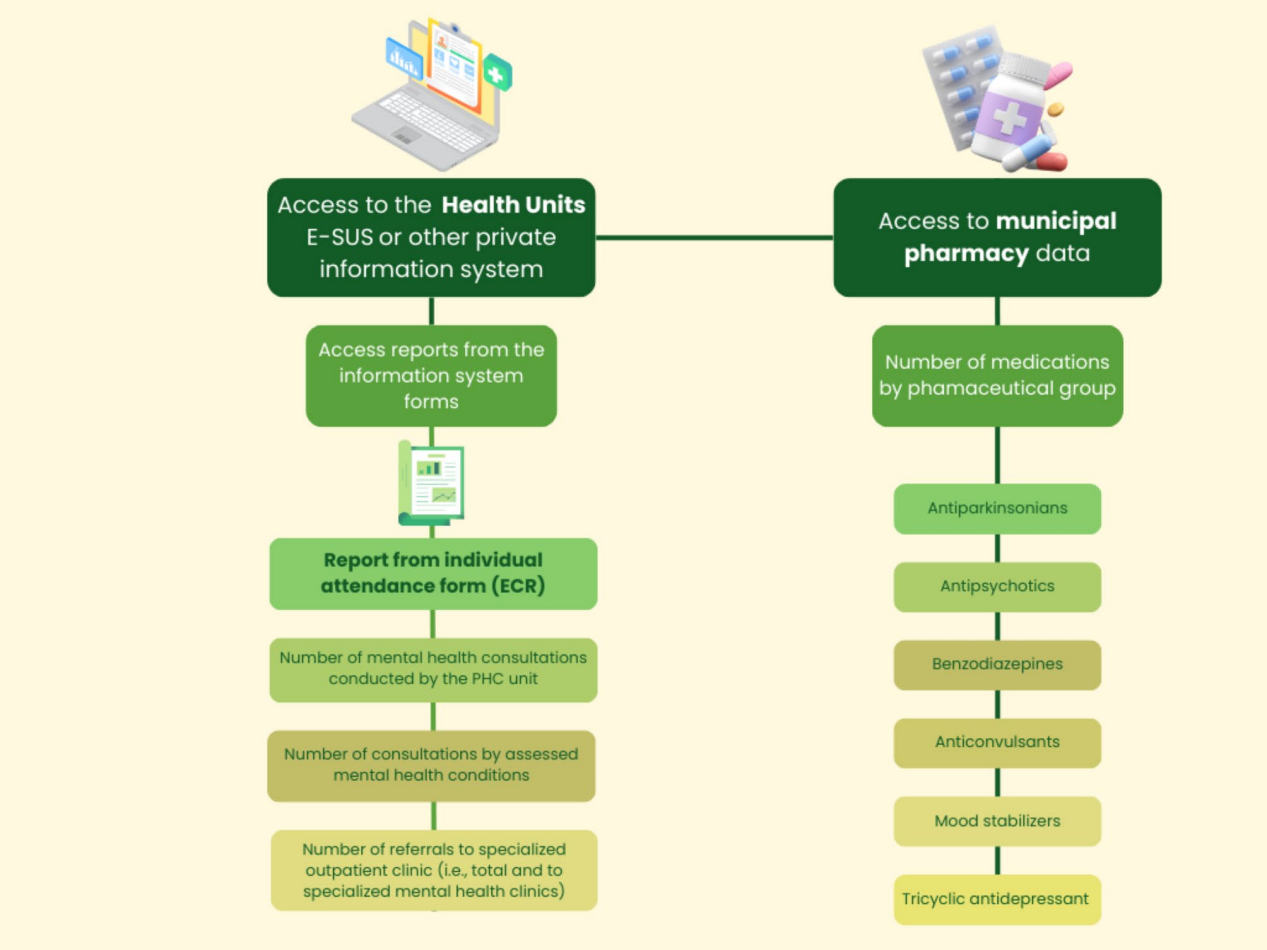
Description of data collection sources Source: The authors PHC: primary health care; ECR: Electronic Citizen Record

At both times, two trained researchers visited all sites from intervention and control groups and collected data from local sources within each participating health unit (or municipality health departments). For data collection, the same instrument used to systematize the retrieval of aggregated data was used for the calculation of MH care process-related indicators at the PHC unit or municipality level at the two data collection periods. MH care process indicators were chosen according to indicators suggested by the Brazilian technical note for mental health care in PHC [19] and considering data possible to be obtained from PHC units and municipal health management routines. The instrument is a spreadsheet with fields for recording a numerator and denominator. Agregated data were manually entered into this structured spreadsheet. The process to extract data from official records were tailored to reflect the specific context of each unit (Fig. 1).

In order to calculate the percentage of consultations using the mental health International Classification of Diseases (ICD) and International Classification of Primary Care (ICPC) classification, we collected aggregated data from reports extracted from the Electronic Citizen Records (ECR) database referring to the total number of consultations with mental health ICD and ICPC records (numerator) and total number of individual consultations (denominator) during the last six months. Regarding the percentage of PHC referrals to specialty mental health care, we collected (i.e., also from reports extracted from ECR database taking into account the total for the last six months) the number of consultations through information system records that resulted in referrals from PHC to a Specialized Mental Health Care unit (numerator) and the total number of referrals completed by the unit (denominator). Concerning the referral rate to specialized mental health care per 100 consultations with an MH ICD and ICPC classification, the following calculation was applied: (number of referrals to specialized mental health care/number of consultations with a mental health ICD/ICPC classification) × 100.

Finally, as one of the expected effects from SMAPS is to provide capacity building for PHC non-specialists to identify and manage MH priority conditions, indicators that could point to a possible change at the pattern psychotropics delivery, representing a proxy of the qualification of this process were considered. To estimate the percentage of medications delivered for PHC stratified by pharmaceutical groups (benzodiazepines, antipsychotics, mood stabilizers, anticonvulsants, and antidepressants) relative to the total psychotropics, the search strategy involved direct access to the list of psychotropic drugs available at the municipal pharmacy and also by accessing information systems with the application of filters for psychotropic drugs within a given month (July was considered at pre and post-intervention periods). After verifying the volume delivered for PHC, the following calculation was applied: (number of psychotropic drug units delivered by pharmaceutical group/number of psychotropic drug units delivered) × 100. Units were accounted by blister pack units (in the case of medicines in tablet form) or by vials/ampoules (in the case of medicines in liquid form).

More details regarding MH care indicators collection and calculation are provided in Fig. 1; Table 2.

**Table 2:** Description of Primary Health Care (PHC) units and municipalities indicators, SMAPS Project, Brazil, 2022‒2023

Indicator (numerator/denominator)	Source	Reference period	Coverage
**Percentage of consultations with a mental health ICD/ICPC record** (number of consultations with mental health ICD/ICPC record/total number of individual consultations)	ECR — individual attendance form	Six-month totals (January to July) ¹	PHC unit
**Percentage of PHC referrals to specialty mental health care** (number of referrals to specialty mental health care/total number of referrals completed by the unit)	ECR — individual attendance form	Six-month totals (January to July) ¹	PHC unit
**Rate of referrals to specialty mental health care per 100 consultations with MH ICD/ICPC record** (number of referrals to specialty mental health care/number of consultations with MH ICD/ICPC record) × 100	ECR — individual attendance form	Six-month totals (January to July) ¹	PHC unit
**Percentage of benzodiazepines dispensed for PHC in relation to psychotropic drugs total** (number of benzodiazepines units delivered for PHC/number of psychotropic drug units delivered) × 100)	Pharmacy report	July	Municipal
**Percentage of antipsychotics**,** mood stabilizers**,** anticonvulsants**,** and antidepressants medications dispensed for PHC in relation to psychotropic drugs total** (number of antipsychotics, mood stabilizers, anticonvulsants, and antidepressants medications units delivered for PHC/number of psychotropic drug units delivered) × 100)	Pharmacy report	July	Municipal

Source: The authors

^1^Data for 2022 were collected from January to June, whereas data for 2023 were collected from February to July

ICD: International Classification of Diseases; ICPC: International Classification of Primary Care; PHC: primary health care; ECR: Electronic Citizen Record

### Data analysis

The use of similarity criteria in the selection of control group PHC units reinforces the possibility that the analysis of differences between means of MH care indicators actually reflect the effects of the intervention. It is important to note that the last data collection point refers to July 2023, when the SMAPS Project intervention was still underway. Thus, the interpretation of the collected data is based on the perspective that the effects of the SMAPS Project are still preliminary and may undergo changes.

**Fig. 2:**
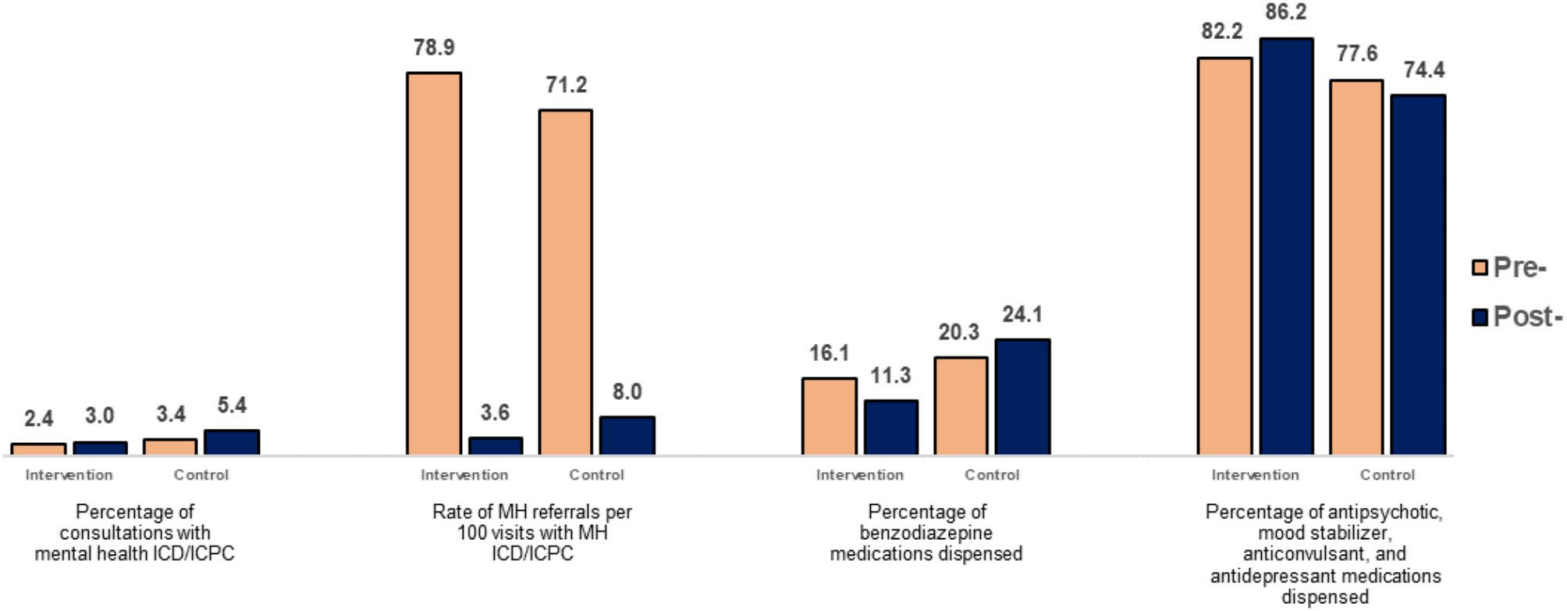
Mean values for mental health care indicators from intervention and control units by data collection period. SMAPS Project, Brazil, 2022‒2023 Source: The authors. ‘Pre-’ indicates that information was collected before the intervention and ‘Post-’ indicates that information was collected one year after the start of the intervention. ICD: International Classification of Diseases; ICPC: International Classification of Primary Care

MH care process indicators were presented for intervention and control sites by each Health Region individually (Tables 4 and 5). Also, data analysis was performed using descriptive statistics at the PHC unit or municipality level, allowing for the description of indicators in the two groups (intervention and control) at two periods (at the start of the intervention and one year after the start of the intervention) (Fig. 2). We performed a Mann-Whitney U test to assess the statistical significance of the difference in the distributions of the time variation in MH care indicators between the intervention and control groups. All analyses were performed using R statistical software (v4.3.0; R Core Team 2023).

## Results

Six intervention and 12 control PHC units participated in the study. The characteristics of the participating PHC units are described in Table 3. Table 4 shows the mean values of the indicators analyzed for intervention and control health units.

**Table 3:** Descriptive characteristics of Primary Health Care units (PHCu) and municipalities participating in the study. SMAPS Project, Brazil, 2022‒2023

Health Region	Unit	PHC coverage (Dec/20)	Projected municipality population (2021)	*N*. of linked records (Jan/22)	*N*. of FHS teams (May/22)
Health Region 1 (Northeast)	Intervention municipality 1 (PHCu -i 1)	100.0%	11,451	6,650	2
	Control municipality 1 (PHCu -c 1)	100.0%	171,317	4,969	2
	Control municipality 1 (PHCu -c 2)	100.0%	171,317	5,092	2
Health Region 2 (North)	Intervention municipality 2 (PHCu -i 2)	79.5%	104,517	7,678	2
	Control municipality 2 (PHCu -c 3)	77.6%	131,026	8,403	2
	Control municipality 2 (PHCu -c 4)	77.6%	131,026	12,914	3
Health Region 3 (North)	Intervention municipality 3 (PHCu -i 3)	94.2%	37,098	7,456	2
	Control municipality 3 (PHCu -c 5)	100.0%	18,165	4,209	2
	Control municipality 3 (PHCu -c 6)	100.0%	18,165	3,041	2
Health Region 4 (Central-West)	Intervention municipality 4 (PHCu -i 4)	100.0%	28,360	3,656	1
	Control municipality 4 (PHCu -c 7)	100.0%	25,597	3,154	1
	Control municipality 4 (PHCu -c 8)	100.0%	25,597	6,579	1
Health Region 5 (Central-West)	Intervention municipality 5 (PHCu -i 5)	100.0%	10,961	3,958	1
	Control municipality 5 (PHCu -c 9)	100.0%	10,680	4,278	1
	Control municipality 6 (PHCu -c 10)	100.0%	10,120	3,195	1
Health Region 6 (Central-West)	Intervention municipality 6 (PHCu -i 6)	93.7%	28,518	3,140	1
	Control municipality 7 (PHCu -c 11)	89.6%	27,365	3,834	1
	Control municipality 8 (PHCu -c 12)	94.9%	31,909	5,891	1

Source: The authors. PHC coverage represents the coverage of the primary health care network in the municipality of the basic health unit. PHC coverage was obtained from the Primary Care e-Manager of the Primary Health Care Department. The projected population for the municipalities was obtained from estimates from the Brazilian Institute of Geography and Statistics (BIGS). The number of records linked to the basic health unit was retrieved from the Health Information System for Primary Care (HISPC). The number of Family Health Strategy (FHS) teams was retrieved from the National Registry of Health Establishments (NRHE). Information on psychologists, community health agents (CHA), and temporary workers was retrieved from EFTC. Employees with fixed-term contracts, commissioned positions, scholarship holders, and freelancers were considered temporary workers for the analysis

**Table 4:** Description of mental health care indicators accumulated for six months at pre- and post- intervention. SMAPS Project, Brazil, 2022‒2023

Health Region	Unit	Number (%) of consultations						Number (%) of referrals						Rate of referrals to specialty MH care per 100 visits with MH ICD/ICPC		
		Pre-			Post-			Pre-			Post-			Pre-	Post-	
		Total	With MH ICD/ICPC (%)		Total	With MH ICD/ICPC (%)		Total	To specialty MH care (%)		Total	To specialty MH care (%)				
Health Region 1 (Northeast)	Intervention municipality 1 (PHCu -i 1)	9235	11 (0.1)		6687	103 (1.5)		945	242 (25.6)		408	26 (6.4)		2200.0	25.2	
	Control municipality 1 (PHCu -c 1)	1902	49 (2.6)		1664	86 (5.2)		129	5 (3.9)		38	0 (0.0)		10.2	0.0	
	Control municipality 1 (PHCu -c 2)	2049	6 (0.3)		2480	42 (1.7)		245	6 (2.4)		95	0 (0.0)		100.0	0.0	
Health Region 2 (North)	Intervention municipality 2 (PHCu -i 2)	4956	226 (4.6)		3142	161 (5.1)		503	0 (0.0)		299	1 (0.3)		0.0	0.6	
	Control municipality 2 (PHCu -c 3)	1582	54 (3.4)		1901	44 (2.3)		1223	54 (4.4)		215	6 (2.8)		100.0	13.6	
	Control municipality 2 (PHCu -c 4)	5820	300 (5.2)		2339	120 (5.1)		1182	300 (25.4)		231	8 (3.5)		100.0	6.7	
Health Region 3 (North)	Intervention municipality 3 (PHCu -i 3)	4640	218 (4.7)		7302	343 (4.7)		417	0 (0.0)		803	2 (0.2)		0.0	0.6	
	Control municipality 3 (PHCu -c 5)	1423	227 (16.0)		2420	383 (15.8)		1698	238 (14.0)		376	3 (0.8)		104.8	0.8	
	Control municipality 3 (PHCu -c 6)	1182	68 (5.8)		2454	159 (6.5)		963	95 (9.9)		196	32 (16.3)		139.7	20.1	
Health Region 4 (Central-West)	Intervention municipality 4 (PHCu -i 4)	4358	50 (1.1)		4426	80 (1.8)		4417	0 (0.0)		88	0 (0.0)		0.0	0.0	
	Control municipality 4 (PHCu -c 7)	6091	176 (2.9)		4799	74 (1.5)		520	161 (31.0)		126	36 (28.6)		91.5	48.6	
	Control municipality 4 (PHCu -c 8)	12,775	15 (0.1)		12,539	37 (0.3)		120	33 (27.5)		74	23 (31.1)		220.0	62.2	
Health Region 5 (Central-West)	Intervention municipality 5 (PHCu -i 5)	2518	109 (4.3)		2820	137 (4.9)		2371	181 (7.6)		273	0 (0.0)		166.1	0.0	
	Control municipality 5(PHCu -c 9)	1612	125 (7.8)		4375	858 (19.6)		‒	‒		‒	‒		‒	‒	
	Control municipality 6 (PHCu -c 10)	2093	30 (1.4)		3611	50 (1.4)		119	0 (0.0)		36	0 (0.0)		0.0	0.0	
Health Region 6 (Central-West)	Intervention municipality 6 (PHCu -i 6)	2971	78 (2.6)		3774	10 (0.3)		382	121 (31.7)		376	1 (0.3)		155.1	10.0	
	Control municipality 7 (PHCu -c 11)	2937	302 (10.3)		2905	292 (10.1)		345	17 (4.9)		291	13 (4.5)		5.6	4.5	
	Control municipality 8 (PHCu -c 12)	2335	52 (2.2)		2820	226 (8.0)		162	0 (0.0)		241	1 (0.4)		0.0	0.4	

Source: The authors. The ‘Pre-’intervention started in January 2022. The ‘Post-’ intervention started in February 2023. ICD: International Classification of Diseases; ICPC: International Classification of Primary Care; MH: mental health; PHC: primary health care. PHCu-c 9 data are not available for the referrals indicator

**Table 5:** Description of indicators for psychotropic medications dispensed in pre- and post- intervention by municipality. SMAPS Project, Brazil, 2022‒2023

Health Region	Municipality	Percentage of benzodiazepine medications dispensed for PHC			Percentage of antipsychotics, mood stabilizers, anticonvulsants, and antidepressants medications dispensed for PHC	
		Pre-	Post-		Pre-	Post-
Health Region 1 (Northeast)	Intervention municipality 1	30.3%	15.2%		69.7%	81.9%
	Control municipality 1	14.1%	5.1%		83.0%	94.9%
Health Region 2 (North)	Intervention municipality 2	17.2%	1.8%		82.7%	94.0%
	Control municipality 2	9.0%	45.9%		88.3%	53.4%
Health Region 3 (North)	Intervention municipality 3	3.7%	10.4%		94.5%	86.8%
	Control municipality 3	15.8%	14.2%		76.8%	80.2%
Health Region 4 (Central-West)	Intervention municipality 4	13.1%	13.3%		84.7%	84.6%
	Control municipality 4	18.8%	18.3%		79.1%	79.8%
Health Region 5 (Central-West)	Intervention municipality 5	17.0%	9.6%		80.4%	89.5%
	Control municipality 5	43.8%	35.2%		55.3%	64.8%
	Control municipality 6	15.4%	27.5%		84.0%	69.3%
Health Region 6 (Central-West)	Intervention municipality 6	15.4%	17.5%		80.9%	80.6%
	Control municipality 7	34.9%	35.7%		65.1%	64.3%
	Control municipality 8	10.8%	11.1%		89.2%	88.9%

Source: The authors. PHC: primary health care

On average, the percentage of consultations with a mental health ICD or ICPC record seemed to increase in both the intervention and control groups (Fig. 2). In addition, both the rate of referrals per consultation with a mental health ICD or ICPC record and the percentage of total referrals to mental health specialties tended to decline both in intervention and control group PHC units (Fig. 1). When looking into individual results of Health Regions 1, 5 and 6 (Table 4) and means values of intervention and control groups, the reduction in the rate of referrals per 100 consultations with a mental health ICD/ICPC seemed to be higher in SMAPS-participating PHC units (75.3%) than in control PHC units (63.2% reduction) (Fig. 2). Nonetheless, both for consultations and referrals indicators, among our sample of 18 units, differences of mean values variation between the pre- and post-intervention were not statistically significant in distribution at the 5% level when intervention and control groups were compared.

Groups results regarding to the indicators for psychotropic medication delivered for PHC suggested that the percentage of benzodiazepine medications delivered for PHC decreased from 16.1 to 11.3% in intervention group municipalities but increased from 20.3 to 24.1% in control group municipalities (Fig. 2). Conversely, an inverse pattern was observed for antipsychotic, anticonvulsant, and antidepressant medications: dispensation between the two assessment times increased from 82.2 to 86.2% in intervention group municipalities and declined from 77.6 to 74.4% in control group municipalities (Fig. 2). Individuals results from Health Regions 2 and 5 are in line with these trends, and individual result from Health region 1 suggest this same pattern in both intervention and control municipalities (Table 5). In our sample of 14 municipalities, differences of mean variation between groups were not statistically significant in distribution at the 5% level.

## Discussion

The present study described MH care process indicators through secondary data collected from PHC services and municipalities routine health records. Results suggested s possible effects of SMAPS on reducing the PHC referrals to specialty mental health services and changing the pattern of delivery of psychotropic medications in PHC, with a reduction in the percentage of benzodiazepine medications and an increase in antipsychotics, antidepressants, and anticonvulsants among the municipalities participating in the project. Nonetheless, none of the differences were statistically significant, probably given the small sample of PHC services and municipalities of our pilot study.

It is noteworthy that the indicators of processes of MH care in PHC used could be calculated from records available in information systems from other Brazilian municipalities. The choice for existing sources of data instead of applying new tools considered the importance of proposing and evaluating metrics that can be incorporated into the work processes of PHC professionals and managers, considering the scarcity of evidence on monitoring and evaluating MH care in the Brazilian scenario [17, 20].

Coelho et al. (2023) [17] evaluated the implementation of PSCN in a Brazilian state, analyzing the structure and activities that constitute the complex range of actions necessary for the satisfactory provision of community psychosocial care. These authors identified low implementation of evaluative criterion ‘Mental health care evaluation and monitoring actions at the municipal level’. This finding emphasizes the urgency to incorporate and qualify the culture of recording, monitoring, and evaluating mental health care indicators to generate scientific evidence and use it for decision-making aligned with the needs of the population.

When considering the applicability of the findings of the current study in the real world, we highlight its potential to equip professionals, managers, and decision makers in the incorporation and monitoring of MH indicators based on systematized national sources, aligned with the resources available in the PHC services and, thus, replicable on a large scale. Moreover, it is known that efforts aimed at developing evaluation processes that take advantage of documented information available in the daily routines of healthcare teams can contribute to the sustainability of these propositions [21].

Although not statistically significant the results regarding a possible effect of SMAPS on the psychotropic medications delivery for PHC suggest improved qualification of the drug therapy offered in PHC. The reduction in the delivery of benzodiazepines concomitantly with an increase in the percentage of antidepressants, antipsychotics, anticonvulsants, and mood stabilizers delivered in PHC was observed among the municipalities in the intervention group from some Health Regions. This result is in line with the guidelines recommended by the WHO [22] and may be a reflection of the continuing education that was delivered during the intervention, through training, workshops, and routine case discussions by a multidisciplinary team. It should be noted that the percentage of benzodiazepines dispensed in relation to other classes of medications supports the findings of a previous study [23], often reflecting inadequate prescribing practices. In addition, Brazilian studies reveal that psychotropic use is inversely related to income level independently of the presence of common mental disorders [24], in particular this association was found to antidepressants but not with benzodiazepine [12]. The last study also indicates a pattern of misuse, i.e. antidepressants are probably underused for mental disorders, and benzodiazepines are probably overused for mental disorders [12]. One important issue related to this scenario regards on challenges in diagnosing and managing mental health issues reported by general practitioners in PHC [25].

The SMAPS project seeks to address these issues by improving the identification and treatment of mental health conditions at PHC the context of the Brazilian public health system (SUS), to reduce disparities and contribute to equitable use of psychotropic medications. The use of aggregated data to evaluate changes in the pattern of psychotropic drug delivery provides a comprehensive overview that could help to identify trends over time. Understanding shifts in delivery patterns could support monitoring of health policies and prescription practices without the need for detailed individual consumption data, thus representing possible evidence to decision-making on resource management.

The possible result of the effect of SMAPS in reducing referrals to specialty mental health care may reflect the alliance of management and care professionals to create spaces for joint discussion to conduct the cases, increasing clarity on strategies for collaborative care practice, case coordination, and longitudinal monitoring [26, 27]. Moreover, it may reflect the results of the joint effort in replicating training for the use of mhGAP-IG, which disseminated knowledge, skills, and attitudes specific to mental health care in PHC.

It is noteworthy that at Health Regions 1 and 6 referrals to specialty mental health care declined both in intervention and control groups. Given that the intervention supports state health governments, it is plausible that the effects of SMAPS may have disseminated among the control units through state-level initiatives, potentially accounting for some of the observed positive changes in the indicators of referrals to specialty mental health care and percentage of consultations with a mental health ICD or ICPC record in this group. Furthermore, the selection of units in both intervention and control groups was based on their prior adherence to the HCP methodology. This may represent a significant factor influencing the organization of work processes in PHC, in addition to the other unexamined external factors involved.

It is worth to highlight that Health Regions 1 and 5 showed better results regarding the pattern of psychotropic delivery and referrals to specialty mental health care. We suggest that it could be related to the better performance of the intervention, estimated by the iHCP-Performance, i.e. both Health Regions pointed the highest score in 2023 (9.0).

Despite the substantial results, it is important to highlight that, when analyzing the rate of referrals to specialty mental health care, we identified a weakness in the calculation of the indicator in some intervention and control units: there were significantly larger numbers of referrals to specialty mental health care compared to the number of consultations with a mental health ICD/ICPC record. This result may reflect the deficiency of ICD/ICPC records during consultations with a mental health care approach, or even suggest a percentage of referrals to specialty care without the intervention being directly linked to the consultation.

One of the main challenges to achieving truly effective mental health care is the need of a paradigm shift towards a multidisciplinary approach aimed at comprehensive care. The importance of ending a mental health consultation in PHC not only with the prescription of medication or referrals, but as the beginning of a more comprehensive conversation, considering the individual’s entire situation, their psychosocial and existential conditions, and therapeutic challenges have been highlighted and are in line with SMAPS. Thus, even though the current study measured a referrals indicator, we highlight the need to define and implement other indicators that demonstrate a significant transition in this care paradigm and the efficiency of PHC in managing and resolving cases of mental disorders.

Among the limitations the lack of statistical significance in the results of the Mann-Whitney U test is likely due to the small sample size, which results in large *p*-values. This does not necessarily indicate the absence of differences in the variation of mental health care indicators between the intervention and control groups. In addition, the still unsatisfactory quality of data recording can affect the accuracy of the information analyzed, as some issues identified in the records, may have compromised the interpretation of some of our results. At this context, using an indicator of psychotropic medication delivery in PHC as a proxy of prescription or consuming might be pointed as a limitation. Data was obtained from the municipality pharmacy report, as the total volume of each type of medication delivered by the municipal pharmacy to PHC. Thus, data regarding individual prescriptions or consuming were not available and was not possible to infer parameters related to dose, duration, or frequency of use.

It is important to highlight the need to consider the time frame of this analysis and contextualize it in relation to the duration of the SMAPS project. Thus, results should be interpreted considering that the last stage of the intervention was just beginning and was expected to be carried out for approximately six months after the completion of the “post” period data collection.

## Conclusion

This is an innovative study that evaluated the effect of the SMAPS project in six Health Regions of three Brazilian states. Our findings may contribute to the formulation of proposals for evaluating mental health care based on data already available from records and reports from health services. The study promotes the qualification of practices for recording and monitoring indicators in PHC, contributing to strengthening mental health care actions and the comprehensive care of other health conditions.

Our pilot study provides important results, especially regarding the referrals to specialty mental health care and the delivery of psychotropic medications, highlighting the need of further research on actions for organizing mental health care in PHC. This work emphasizes the need to improve the quality of health recording in PHC work routines, allowing for the monitoring of indicators of MH care at PHC.

Future studies should work with more PHC units and longer post-intervention intervals to assess the sustainability of the actions. Also, future qualitative evaluations should be done to reveal additional aspects of the organization of the mental health care process in PHC, providing more detailed insights into the effectiveness of the interventions implemented.

## Data Availability

The datasets used and/or analyzed during the current study are available from the corresponding author on reasonable request.
